# Bioactive Silicon Nitride Implant Surfaces with Maintained Antibacterial Properties

**DOI:** 10.3390/jfb13030129

**Published:** 2022-08-27

**Authors:** Ioannis Katsaros, Yijun Zhou, Ken Welch, Wei Xia, Cecilia Persson, Håkan Engqvist

**Affiliations:** 1 Division of Applied Materials Science, Department of Materials Science and Engineering, Uppsala University, 75103 Uppsala, Sweden; 2 Division of Biomedical Engineering, Department of Materials Science and Engineering, Uppsala University, 75103 Uppsala, Sweden; 3 Division of Nanotechnology and Functional Materials, Department of Materials Science and Engineering, Uppsala University, 75103 Uppsala, Sweden

**Keywords:** bioactivity, silicon nitride, surfaces, antibacterial, biomedical

## Abstract

Silicon nitride (Si_3_N_4_) is a promising biomaterial, currently used in spinal fusion implants. Such implants should result in high vertebral union rates without major complications. However, pseudarthrosis remains an important complication that could lead to a need for implant replacement. Making silicon nitride implants more bioactive could lead to higher fusion rates, and reduce the incidence of pseudarthrosis. In this study, it was hypothesized that creating a highly negatively charged Si_3_N_4_ surface would enhance its bioactivity without affecting the antibacterial nature of the material. To this end, samples were thermally, chemically, and thermochemically treated. Apatite formation was examined for a 21-day immersion period as an in-vitro estimate of bioactivity. Staphylococcus aureus bacteria were inoculated on the surface of the samples, and their viability was investigated. It was found that the thermochemically and chemically treated samples exhibited enhanced bioactivity, as demonstrated by the increased spontaneous formation of apatite on their surface. All modified samples showed a reduction in the bacterial population; however, no statistically significant differences were noticed between groups. This study successfully demonstrated a simple method to improve the in vitro bioactivity of Si_3_N_4_ implants while maintaining the bacteriostatic properties.

## 1. Introduction

Silicon nitride (Si_3_N_4_) is a ceramic material that has a long history of being utilized in applications where components are to be exposed to thermally and mechanically demanding environments, such as combustion engines and gas turbines [1]. The main driving forces behind its use are the mechanical properties of the material, resulting from its unique microstructure [2], in combination with its refractory nature as a ceramic material. Eventually, silicon nitride was evaluated as a potential biomaterial. Silicon nitride has indeed proven to be non-toxic both in vitro and in vivo [3,4] and this, in combination with its mechanical properties, has led to its use as an orthopedic implant material [5,6,7]. Today, spinal fusion devices made from silicon nitride have been approved by regulatory agencies for human use [8]. Compared to other common spinal implant materials such as Ti6Al4V and poly(ether ether ketone) (PEEK), it has been found to be more osteogenic both in vitro and in vivo [9,10,11,12] and to have comparable clinical results [13,14]. Furthermore, the surface of silicon nitride materials has been found to be inhibitory to bacterial attachment [15,16,17], and even induce lysis in specific bacterial strains through the formation and elution of ammonia ions when in the presence of water [18].

The ideal spinal fusion material should rapidly form a strong bond with the native bone while shielding the affected area from bacterial infection. Failure to do so might lead to pseudarthrosis or non-union of the affected vertebrae, requiring revision surgery [19]. Pseudarthrosis is responsible for almost a quarter of revision surgeries after attempted fusions in the lumbar spine [20]. Its diagnosis can be challenging, and it can lead to disability, pain, and discomfort for affected patients. While there are many risk factors for pseudarthrosis including patient health, age, and construct length [21] among others, its incidence can be reduced by way of implant material selection.

Indeed, increasing the bioactivity of spinal implant materials can be a way to increase the fusion rate and reduce the incidence of pseudarthrosis [22,23]. The term “bioactivity” has been used to describe the ability of a material to spontaneously form a layer of apatite on its surface after immersion in simulated body fluid [24]. Bioactive biomaterials are hypothesized to more rapidly form a bond with the native bone after implantation, resulting in enhanced implant stability. Researchers have identified a variety of properties that are correlated with bioactivity where the surface charge [25] and roughness [26] of the material are key factors. A negative surface charge at physiological pH leads to a stronger attraction of Ca^2+^ ions to the surface of the material, while rougher materials provide more nucleation sites for apatite.

In Si_3_N_4_, these properties can be affected by thermal and/or chemical surface treatments [27]. Bock et al. [28] utilized a variety of thermochemical surface modifications to alter the surface charge and chemistry of silicon nitride materials in order to examine their effect on properties that directly affect the biological behavior of implants such as surface roughness, charge, and wettability. The authors speculated that from the chosen modifications, heat treatment of silicon nitride samples at 1070 °C for 7 h was particularly promising, as it created a highly negatively charged and hydrophilic surface. Bock et al. [15] compared different modulations of Si_3_N_4_, Ti6Al4V, and PEEK in terms of their bacteriostatic behavior. They found that silicon nitride was clearly bacteriostatic, with as-fired, oxidized, nitrogen annealed and SiYAlON coated samples retaining that property at comparable levels. Finally, Hnatko et al. [29] successfully increased the bioactivity of silicon nitride materials through an oxyacetylene flame treatment that oxidized their surface and created a porous surface layer that promoted apatite formation and cell attachment. 

However, a cost-effective and easy-to-apply modification that could be used to enhance the bioactivity of currently-used silicon nitride implants, without affecting their antibacterial behavior, has yet to be identified. The aim of this study was to enhance the bioactive nature of silicon nitride materials through different surface modifications that could potentially be used post-manufacturing before implants reach the clinic. A secondary aim was to ensure that the surface modifications do not affect the inhibitory bacterial environment the material creates. To achieve these aims, commercially-produced silicon nitride samples were thermally, chemically, and thermochemically treated. Afterwards, the chemical or morphological changes on the silicon nitride surfaces were studied. Finally, the effectiveness of each treatment in terms of increasing the bioactivity and inhibiting bacterial proliferation was evaluated in vitro.

## 2. Materials and Methods

### 2.1. Materials

Two types of Si_3_N_4_ samples were used in this study. Bulk non-porous bars (length = 47 mm, height = 2.9 mm, width = 4 mm) and porous cylinders (ø12.6 mm, height = 10 mm, approximate porosity 70%) were both produced by SinTX Technologies (Salt Lake City, UT, USA) using alumina (6% wt) and yttria (4% wt) as sintering additives. This composition is currently used for the production of Si_3_N_4_ spinal implants [30]. The bulk non-porous samples were used for surface characterization after the surface modifications and the assessment of antibacterial behavior, while the porous samples were utilized to evaluate bioactivity after having their morphology studied.

### 2.2. Surface Modifications

Three different surface modification processes were utilized to enhance the bioactive nature of the material. Before being treated, all samples were washed in consecutive 15-min steps in distilled water and ethanol, and then sonicated and thoroughly dried in a desiccator. The samples were divided into 4 groups. 

The control group consisted of the non-treated (NT) samples. The chemically treated (CT) group consisted of samples that were immersed in falcon tubes containing 50 mL of a 10 M sodium hydroxide (NaOH) solution (pellets, Sigma-Aldrich, St. Louis, MI, USA) for 24 h in an environment heated to 60 °C. To ensure homogenous surface treatment, samples were suspended using fishing line, ensuring they were not in contact with the falcon tube walls. The thermally treated (TT) samples were placed in an induction furnace (Gero CWF, Carbolite Furnaces, Sheffield, UK) and heat treated at a temperature of 1070 °C with a ramping rate of 12 °C/min for 4 h, then left to cool overnight. Finally, the thermochemically treated samples (TCT) were thermally and then chemically treated as described above. 

### 2.3. Material Characterization

To further understand the effect each type of modification had on the materials, a variety of characterization methods were employed. The detailed characterization of the samples post-modification also aimed to ensure that any differences in in vitro bioactivity and antibacterial behavior would not be attributed to any differences between samples other than the ones brought on by the modifications. 

#### 2.3.1. X-ray Diffraction (XRD)

X-ray diffraction (D500, Bruker, Billerica, MA, USA) was used to identify whether any new crystalline phases were formed on the surface of the material after the treatments. The samples were scanned from 10° to 80° with a scanning rate of 0.02 °/s using a Bragg-Brentano configuration and CuKa radiation (λ = 0.15418 nm) [31]. 

#### 2.3.2. X-ray Photoelectron Spectroscopy (XPS)

X-ray photoelectron spectroscopy (Quantera II, Physical Electronics, Chanhassen, MN, USA) was used to further elucidate the chemical composition of the surface of the samples. Survey scans were taken at a pass energy of 140 eV, after which atomic percentage concentrations were calculated from the elemental peak area. Results were averaged after 10 measurement cycles. Prior to measurements, samples were sputtered at 500 V for one minute to remove surface contamination. Ion and electron guns were turned on during measurements to neutralize the surface charge build-up of the non-conductive samples. Data analysis was performed using the MultiPak software (Version 9.6, Physical Electronics, Chanhassen, MN, USA). 

#### 2.3.3. Scanning Electron Microscopy (SEM)

Scanning electron microscopy (SEM 1530, Zeiss, Jena, Germany) was utilized in order to examine the surface of the porous samples after the treatments. Samples were coated with a layer of conductive Au/Pd with a thickness of approximately 10 nm to avoid sample charging. Images of the samples were taken after the surface treatments at an accelerating voltage of 8 kV, and a working distance of approximately 5 mm. Images of the samples after the immersion assay were taken at an accelerating voltage of 5 kV, and a working distance of approximately 5 mm. The adjustment in the accelerating voltage was made as apatite formation increased charging. 

#### 2.3.4. Computed Microtomography Scans (μCT)

Computed microtomography scans (Skyscanner, Bruker) of the porous samples were taken in order to visualize the porous network of the samples and to identify the effect, if any, the surface treatments had on it. Scans were taken at a resolution of 9 μm, with a voltage of 100 kV, a current of 100 μA, an exposure time of 2070 ms and a rotation step of 0.4°. The μCT images were reconstructed with NRecon (Bruker) and post-processed in MATLAB (Version 2021b, MathWorks, Natick, MA, USA). The 3D-models were thresholded with respect to the histogram of grey level value distribution and the porosity was calculated in MATLAB (Version 2021b, MathWorks, Natick, MA, USA) by counting the number of remaining voxels.

### 2.4. Immersion Assay

Porous samples (n = 4) from each group were suspended using a fishing line in Dulbecco’s phosphate-buffered saline (DPBS) (Sigma-Aldrich) in order to avoid preferential apatite deposition on sample surfaces, for a total of 21 days. This method was previously used to evaluate the surface bioactivity of biomaterials [32]. DPBS supplemented with CaCl_2_ and MgCl_2_ was used, as it has a similar ionic concentration to that of blood plasma and can simulate physiological fluids [33]. During the first two weeks of immersion, the solution was not replenished in order to facilitate inductively coupled plasma optical emission spectrometry (ICP-OES) (PerkinElmer ICP-OES, Avio 200, PerkinElmer, Waltham, MA, USA) measurements of calcium ions in the solution. These measurements were taken after 1, 5, 7, and 14 days of immersion as a way to pinpoint both the start and the rate of precipitation. A decrease of calcium in the solution would indicate the formation of precipitates. After the first two weeks of immersion, the DPBS solution was replenished in order to provide a fresh supply of ions to support potential apatite formation. After 21 days of immersion, the samples were removed, washed gently with deionized water, and dried in a desiccator. Cross-sections of the porous samples were examined through SEM to evaluate apatite formation. 

### 2.5. Optical Profilometry

Bacterial and cell attachment have been shown to be significantly affected by the surface roughness of the material on which they are seeded [34,35,36]. The samples used for the bacterial attachment assay were initially polished down to an average surface roughness (Sa) of approximately 5nm. Optical profilometry (ZYGO, Middlefield, CT, USA) was used in order to study the effect of the treatments on the average surface roughness of the samples. A 50× magnification was used to scan a square area (α = 167 μm), with five replicates per measurement. Three measurements were taken per sample, after which the results were averaged.

### 2.6. Bacterial Testing

#### 2.6.1. Bacterial Culture

Gram-positive Staphylococcus aureus bacteria were used for this study. 10 μL of a bacterial suspension was added to 10 mL of sterilized tryptic soy broth (TSB) (Sigma-Aldrich), and the mixture was incubated overnight at 37 °C. Following this, the suspension was centrifuged to isolate the bacteria, which were then resuspended in 10 mL of TSB. Finally, the bacterial solution was diluted to an OD_600_ = 0.2, measured using a UV-VIS spectrophotometer (UVmini-1240, Shimadzu, Kyoto, Japan).

#### 2.6.2. Bacterial Attachment Assay

Polished bulk silicon nitride samples were autoclaved at 120 °C for 20 min to avoid cross-contamination with other environmental bacterial strains. The samples were then placed in sterile ø10 cm Petri dishes (Sigma-Aldrich). 10 μL of the diluted bacterial solution was then placed on the surface of each sample for two hours at room temperature so the bacteria could come in contact and interact with the surface of the material. As a negative control, the same volume of bacterial solution was placed into a 2 mL polypropylene microcentrifuge tube for the same amount of time. Both the petri dishes and the microcentrifuge tube were kept closed, and the bacterial solutions were monitored to ensure the bacterial suspension did not evaporate during the incubation period. When the incubation time elapsed, the samples were placed in 1 mL of DPBS (Sigma-Aldrich) and vortexed for 1 min each so the adherent bacteria could be detached from the surface of the samples. One ml of PBS was added to the bacterial solution being used as a control, which was then also vortexed for 1 min. These bacterial solutions were then diluted tenfold in four steps, with 100 μL of each dilution then plated onto TSB-agar plates. The agar plates were then incubated overnight so bacterial colonies could form and then be counted.

### 2.7. Statistical Analysis

Quantitative data is reported as means ± standard deviations. IBM SPSS Statistics (Version 26, IBM Corp, New York, NY, USA) was used to perform a one-way analysis of variance (ANOVA) with Tukey post-hoc tests to identify significant differences between groups. Welch’s robust test of equality of means combined with Tamhane’s post-hoc test was used when the assumption of homogeneity of variance was violated. A significance level of *p* = 0.05 was set for all tests. 

## 3. Results and Discussion

### 3.1. XRD

**Figure 1:**
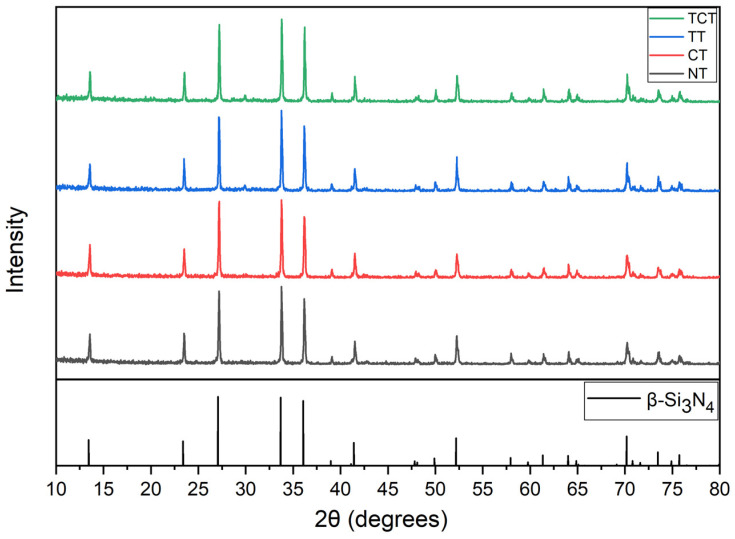
The XRD pattern from samples of all groups are displayed in comparison with the reference pattern of β-Si_3_N_4_ (PDF 01-078-2963), indicating that this was the main crystalline phase present in all groups.

The XRD pattern of all samples can be seen in Figure 1. All samples had the typical peak distribution of β-phase silicon nitride. The samples used in this study, both bulk and porous, were manufactured using thermal processing which ensured the transformation from the α-Si_3_N_4_ to the β-Si_3_N_4_ phase, which is favorable to the mechanical properties of the material [37]. No other crystalline phases were detected in any of the treated samples. However, the potential formation of amorphous sodium silicates in NaOH treated samples, or amorphous silicon dioxide in heat-treated samples, cannot be excluded.

### 3.2. XPS

**Figure 2:**
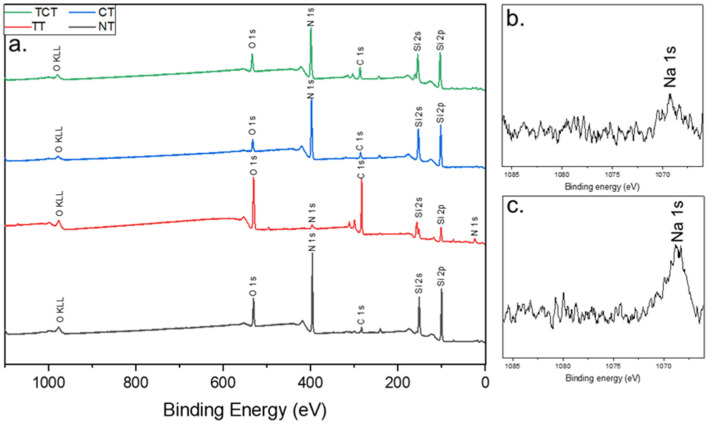
XPS survey spectra, in (**a**), of all samples post-modification showed that the main elements on their surface were mostly the same. However, the difference in peak intensities indicates differences in their amounts. Most notably in thermally treated samples, oxygen peaks were intensified while nitrogen peaks were diminished. Also, both TCT and TT samples had higher amounts of carbon contamination, possibly as a result of the heat treatment. (**b**,**c**) corresponds to the area where the sodium peaks were noticed in thermochemically (**b**) and chemically (**c**) treated samples.

Figure 2 shows that minuscule amounts of sodium were detected on the surfaces of bulk samples. Looking at the atomic concentrations of the surfaces of all groups (Table 1), the thermally treated samples stand out. It is clear that a layer of silicon dioxide was formed, while a very small amount of nitrogen remained present on the surface of the material, as expected from the literature [38]. More interestingly, in the thermochemically treated samples, the NaOH treatment seemed to have etched the samples, removing the oxide layer formed through the heat treatment.

### 3.3. SEM before the Immersion Assay

**Figure 3:**
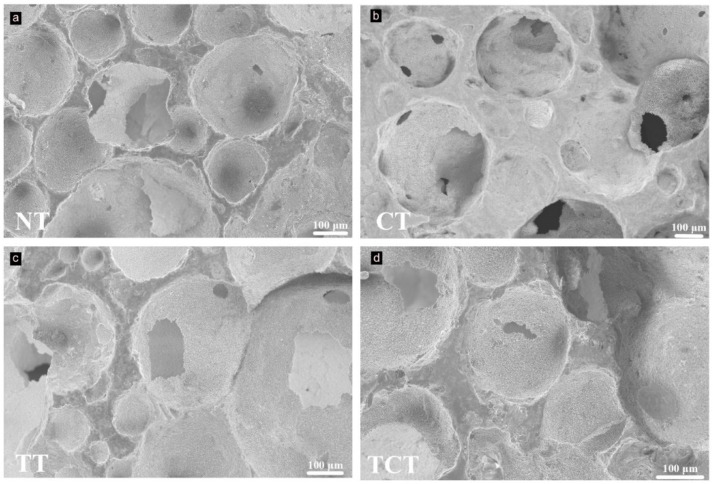
SEM images of the samples after the surface modifications, showing no significant differences in pore size and morphology between (**a**) non-treated, (**b**) chemically, (**c**) thermally, and (**d**) thermochemically treated samples.

SEM images of the samples after the treatments did not show differences in morphology (Figure 3). All samples showcased an extended porous network with pores of varying sizes. No shrinkage was noticed; the heat treatment at 1070 °C without external pressure did not create the necessary diffusion conditions for further consolidation of Si_3_N_4_. 

### 3.4. Computed Microtomography Scans 

**Figure 4:**
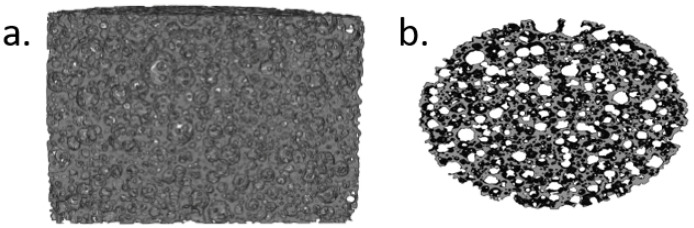
MicroCT 3D reconstructions of (**a**) a longitudinal cross section and (**b**) a transverse slice, visualizing the porous network of samples of all groups. (**a**) has been cropped to exclude artefacts due to sample fixation during scanning and (**b**) has been slightly rotated to enhance visibility.

As evidenced in Table 2, no statistically significant differences (*p* = 0.858) were found in terms of porosity of the samples, which was approximately 70%. The same homogeneity was noticed when examining the CT scans of all groups. The slice (thickness = 270 μm) displayed in Figure 4 confirmed the porous structure observed in the SEM images. Porosity and pore interconnectivity have been found to affect the bioactivity of porous materials [39,40], however, the non-significant differences between groups indicate that any differences in apatite formation do not stem from differences in the porous structure of the samples. Approximating microporosity using μCT is limited in regards to resolution. The resolution of these measurements was around 9.1 μm, meaning that pores below that size could not be accounted for. However, in the case of the presented materials, SEM images confirmed that the material was mainly comprised of pores that, at the very least, were ten times greater that the lowest resolution of the material. 

### 3.5. ICP-OES

**Figure 5:**
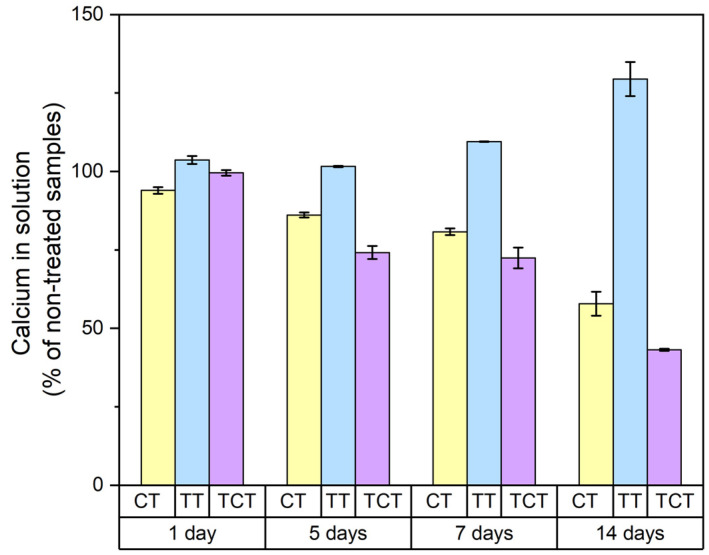
The results of the ICP measurements showing the calcium concentration during the first 14 days of immersion in PBS. A clear decreasing trend was detected for CT and TCT samples throughout the immersion period, indicating calcium precipitation.

As can be seen in Figure 5, the samples that were thermochemically treated showed a decrease in calcium concentration compared to that of non-treated samples as early as the fifth day of immersion. A similar trend was seen in the chemically treated samples, with the decrease in calcium in the solution being more gradual. These results were an indication that the sodium hydroxide treatment was effective in enhancing the bioactivity of porous silicon nitride samples. Thermally treated samples did not show a reduction, and did not significantly diverge from the range of values of DPBS. 

### 3.6. Apatite Formation

**Figure 6:**
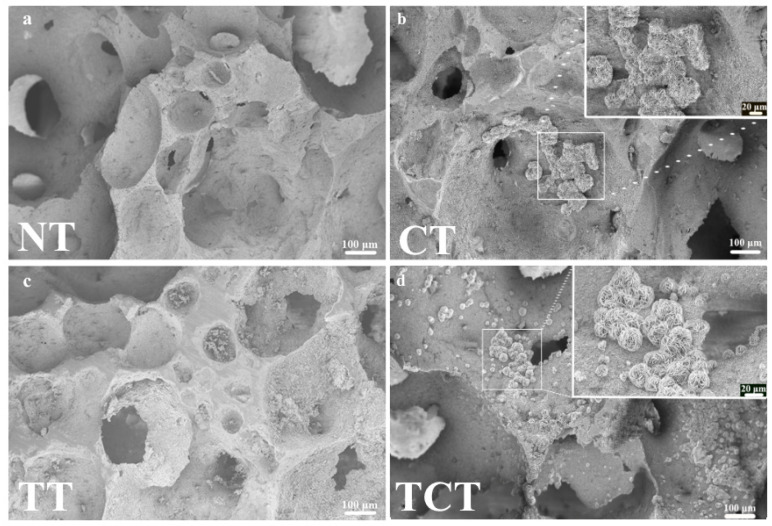
SEM images taken at 200× magnification for samples of all groups after 21 days of immersion. Apatitic flakes were evident for CT (**b**) and TCT (**d**) samples but not in the NT (**a**) and TT (**c**) ones. In (**c**) some remaining debris from sample preparation can be seen.

SEM images after immersion confirmed the results of the ICP measurements (Figure 6). As far as the samples of group TCT were concerned, a large number of spheres showcasing the characteristic apatitic plate-like structures were developed throughout the surface of the samples. Looking at the CT samples, their surface was similar, but with less pronounced apatite formation. Those structures were not noticed in the NT and TT samples, in accordance with the ICP-OES results. 

**Figure 7:**
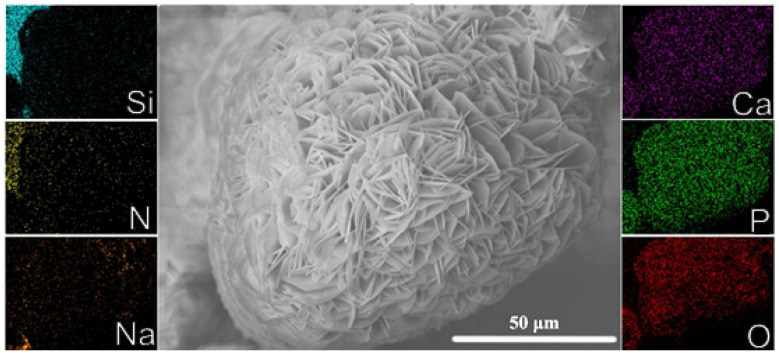
EDS analysis confirmed that the apatite-like structures mainly consisted of Ca, P, and O. Traces of sodium were identified throughout the surface of the thermochemically treated materials.

SEM analysis indicated that the chemical and thermochemical treatment did have the expected effect. Between the two, the TCT samples had a surface with a denser network of the characteristic plate-like structures of apatite (Figure 7). In the non-treated and thermally treated samples, no apatitic flakes were detected. The heat treatment resulted in an oxidized surface that did not seem to have any enhancing effect in terms of apatite formation. The only indication of precipitation were amorphous layers in their forming stages in parts of the material.

The initial hypothesis of the study was that surface charge would be the main mechanism behind any increase in bioactivity, however, the results indicate that another mechanism was at play. The surface of silicon nitride materials in wet environments is comprised of silanol (Si-OH) and amine (Si-NH_2_) groups. The ratio of silanol to amine groups is inversely proportional to the surface charge of the material at physiological pH. The thermally treated samples had a surface dominated by silicon oxynitride and, consequently, a highly negative charge at physiological pH [28]. Surprisingly, no specific enhancement resulted from the thermal treatment. Instead, the chemical and thermochemical treatments clearly enhanced bioactivity. The results of the ICP-OES and qualitative assessment of the SEM images showed that the thermochemically treated samples had more calcium phosphates precipitated on their surface. This led to the conclusion that sodium hydroxide was a significant factor in the increase of bioactivity. While sodium has not been found to be essential for apatite formation in bioactive glasses [41], this study clearly indicates that NaOH treatment enhances apatite formation. In their study on the mechanism of apatite formation on sodium silicate glasses, Hayakawa et al. [42] identified sodium binding in silicon tetrahedra as being crucial to the formation of negatively charged sites. They found that the dissolution of calcium silicates creates sites for the nucleation and crystallization of apatite. XPS and EDS analysis showed the clear retention of sodium throughout the porous structure of the materials. Thus, the effectiveness of the NaOH treatments seems to stem from the dissolution of calcium silicates creating negatively charged vacancies, suitable for calcium precipitation. It could be hypothesized that the general surface charge of the material plays a lesser role in the nucleation of apatite than localized negatively charged sites. 

**Figure 8:**
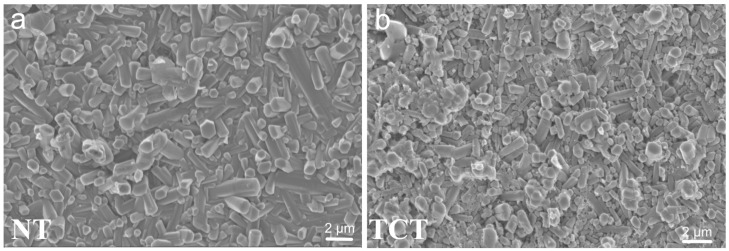
SEM images taken at a 10,000× magnification revealed an increase in grain size in the thermochemically treated samples (**b**) when compared to the non-treated samples (**a**), as a result of the heat treatment.

Finally, the increased bioactivity of TCT samples in comparison to CT samples may be explained by the grain growth noticed in the TCT samples, due to the heat treatment (Figure 8). The elongation of the needle-like β-Si_3_N_4_ grains created a rough surface that created a surface morphology favorable for the precipitation of calcium phosphates.

The results of the immersion assay should be interpreted with caution. Kokubo et al. [32] reported that a surface layer of calcium phosphate formed on Bioglass, which was speculated to be the cause of a faster and stronger bond with the natural bone after in vivo implantation through preferential osteoblast attachment and proliferation on that apatite layer. Consequently, it was proposed that the occurrence of spontaneous apatite formation on materials immersed in solutions with ion concentrations similar to that of the human blood plasma is a valid indication of their in vivo behavior. Since then, there has been an interesting debate on the validity of these immersion assays as predictors of in vivo behavior. Bellucci et al. [43] examined the biological behavior of commercial Bioglass and Bioglass composites using immersion in SBF as well as cell attachment, viability, and proliferation assays, finding that the two sets of methods could give contradicting results and, thus, immersion studies could be misleading. Furthermore, Bohner and Lemaitre [44] also stated that the results of such assays cannot be standalone predictors of natural bone-bonding as they can indicate false-positive or negative results. With this debate in mind, an immersion in DPBS was utilized in this study as a first estimation of bioactive behavior, with further studies needed to validate these results and approximate in-vivo behavior.

### 3.7. Optical Profilometry

**Figure 9:**
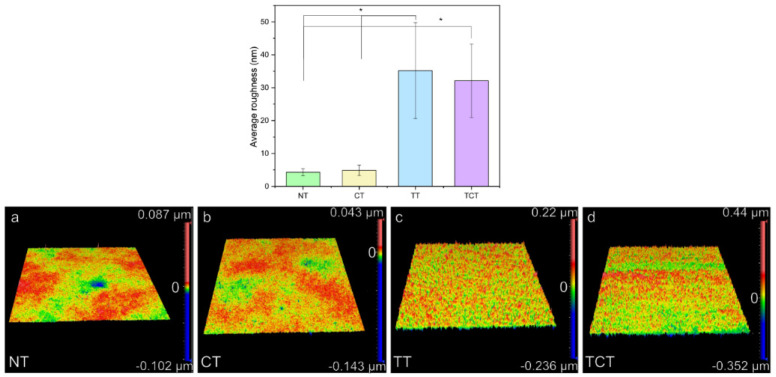
The results of the optical profilometry measurements showed a statistically significant difference between heat-treated (TT, TCT) and non-heat-treated samples (NT, CT). Compared to the (**a**) non-treated and (**b**) chemically treated ones, evidenced by the larger red peaks on the surface reconstructions, the (**c**) thermally treated and (**d**) thermochemically treated samples had rougher surfaces.

Heat treating the samples led to grain growth that due to the needle-like morphology of the β-grains of the material increased surface roughness in Figure 9. As expected, heat-treated samples showed an almost ten-fold increase in average surface roughness compared to the non-heat-treated ones. Differences between the TT and TCT groups versus the NT and CT groups were statistically significant (* *p* ≤ 0.001 for all). No statistically significant differences were found between the NT and CT groups (* *p* = 0.966), nor between the TT and TCT groups (* *p* = 1.0). 

### 3.8. Colony Forming Unit Assay

**Figure 10:**
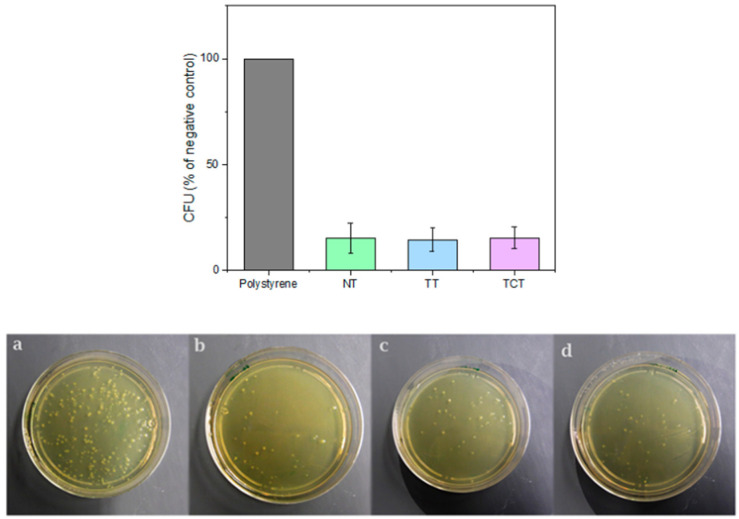
Top: The colonies formed on samples expressed as a percentage of the negative control. Bottom: Indicative images of colonies formed by bacteria after being in contact with: (**a**) Polystyrene, (**b**) Non-treated Si_3_N_4_, (**c**) Thermally treated Si_3_N_4_, (**d**) Thermochemically treated Si_3_N_4_. A clear reduction in bacterial population can be noted for all silicon nitride materials.

The samples that were thermochemically treated showed the highest bioactivity and were therefore selected to be compared with the non-treated ones in terms of their antibacterial behavior. As the difference between the surface roughness of the two groups was significant, samples of the TT group were tested as well in an effort to account for the effect of the increased surface roughness. Figure 10 displays the amount of viable colony-forming units after contact with the tested groups and the controls. The results show a clear inhibitory effect by the material, with the number of colony-forming units developed on the samples being approximately 15% of the negative control. However, no statistically significant difference (*p* = 0.879) was detected between the untreated and treated silicon nitride samples. 

Nitrogen, through the surface groups formed by it, plays a significant role in the antibacterial behavior of the material [45]. Thus, it is not surprising that all materials retained bacteriostaticity as nitrogen was present on all of their surfaces, though in different amounts. Again, the behavior of the thermally treated samples is interesting. XPS analysis (Table 1) showed that the surface of these samples contains very low amounts of nitrogen. Nevertheless, the samples did not significantly diverge from other groups in terms of their interaction with the bacteria. This could be an indication that even surfaces with low amounts of nitrogen can exhibit antibacterial behavior. The results of the in vitro CFU assay are in agreement with a study by Bock et al. [15] that examined the effect of surface modifications on the bacteriostatic properties of silicon nitride, showing that heat-treated samples showed comparable antibacterial behavior to non-heat-treated ones. Furthermore, in a recent study, Kushan Akin et al. [46] investigated the effect of the amount of nitrogen in oxynitride glasses on their antibacterial behavior. They found that increasing nitrogen content did not result in enhanced antibacterial behavior, as there are a plethora of factors governing the material-pathogen interaction. Such factors can include surface topography and roughness, as well as surface charge and wettability.

## 4. Conclusions

This study successfully demonstrated a simple way through which Si_3_N_4_ implants can become more bioactive in vitro. A thermochemical surface treatment resulted in more apatite nucleation sites without influencing the bacteriostatic nature of the surface of the material. A higher rate of osteointegration could be especially important for spinal fusion implants, the current main biomedical application of Si_3_N_4_, as it would improve implant stability and increase fusion rates. 

## Figures and Tables

**Table 1:** Atomic concentration of the main elements comprising the surface of the samples of each group (all atomic concentration values have been rounded to integers).

Atomic Concentration (%)					
Group	Si	N	O	Si/N	Si/O
Non-treated	40	39	16	1	2.5
Chemically treated	40	44	7	0.9	5.7
Thermally treated	15	2	38	7.5	0.4
Thermochemically treated	35	38	12	0.9	2.9

**Table 2:** The average overall porosity of the porous samples of all groups after the modifications, as estimated by μCT.

Average Porosity (%)	
Non-treated	68.7 ± 5.1
Chemically treated	70.2 ± 3.3
Thermally treated	70.3 ± 0.8
Thermochemically treated	69.2 ± 1.5

## Data Availability

Not applicable.
